# Active Student Participation May Enhance Patient Centeredness: Patients' Assessments of the Clinical Education Ward for Integrative Medicine

**DOI:** 10.1155/2013/743832

**Published:** 2013-03-19

**Authors:** Christian Scheffer, Diethard Tauschel, Melanie Neumann, Gabriele Lutz, Maria Valk-Draad, Friedrich Edelhäuser

**Affiliations:** ^1^Integrated Curriculum for Anthroposophic Medicine (ICURAM), Center for Integrative Medicine, Integrative and Anthroposophic Medicine, Faculty of Health, Witten/Herdecke University, School of Medicine, Alfred-Herrhausen-StraBe 50, 58448 Witten, Germany; ^2^Center for Educational Research, Faculty of Health, Witten/Herdecke University, 58448 Witten, Germany; ^3^Department of Internal Medicine, Clinical Education Ward for Integrative Medicine (CEWIM), Gemeinschaftskrankenhaus Herdecke, 58313 Herdecke, Germany; ^4^Faculty of Health, Witten/Herdecke University, 58448 Witten, Germany; ^5^Department of Early Rehabilitation, Gemeinschaftskrankenhaus Herdecke, 58313 Herdecke, Germany

## Abstract

*Objectives*. To examine the impact of active student participation on quality of care in an integrative inpatient setting. *Methods*. Over a two-year period, we surveyed all patients treated on the Clinical Education Ward for Integrative Medicine (CEWIM), where final-year medical students are integrated into an internal medicine ward complementing conventional medicine with anthroposophic medicine. Patients treated on the regular wards of the same internal medicine department served as the control group (CG). General quality of care was studied with the Picker Inpatient Questionnaire, physician empathy with the Consultation and Relational Empathy measure, and patient enablement with the Patient Enablement Index. ANCOVA was used to control for covariates while examining significant differences between both patient groups. *Results*. Comparison of the CG wards and the CEWIM revealed no significant differences in medical treatment success. The CEWIM, however, achieved better results for physician-patient interaction, physician empathy, and patient enablement. Eighty Percent of the CEWIM patients rated student participation as positively impacting quality of care. *Conclusion*. Our results indicate that incorporating students in an integrative healthcare setting may result in greater patient centeredness. Further studies are needed to determine whether this is due to organizational advantages, students' empathic activity, the impact of teaching, or learner-teacher interaction.

## 1. Introduction

Over the last two decades, increased attention has been given to the concept of active learner participation in the so-called “community of practice” [1, 2]. Studies have suggested that apart from the explicit knowledge typically taught in the classroom, learners must also acquire certain implicit skills and knowledge within their future workplace. In addition, active participation in patient care in particular seems to play a crucial role in the acquisition of professional and interpersonal competencies and the development of a sense of role identity [3]. 

In the field of medicine, active student participation (ASP) typically takes place during clinical clerkships and final-year rotations. However, from different studies, we know that participation in clinical rotations alone does not necessarily automatically result in the professional development of learners. Instead, it is the degree of active participation (as opposed to being involved in primarily passive learning experiences) together with certain qualities of the learning environment that determine the success of clinical education [3, 4]. In Germany, for example, medical students are usually required to do three 16-week final-year rotations in internal medicine, surgery, and one field of their choice. However, as in other countries [4, 5], the educational quality of these rotations has been criticized as being suboptimal. Students tend to be provided with mere passive experiences rather than actively participating in patient care. They are all too often assigned routine tasks, and do not receive sufficient clinical supervision. These shortcomings have been shown to lead to insufficient training of independent patient management and to feelings of uncertainty among medical students [6]. 

In response to these deficits and in order to offer students a structured clinical learning environment tailored to their learning needs, the Clinical Education Ward for Integrative Medicine (CEWIM) was established in 2007 and implemented into one of the wards of the Department of Internal Medicine at the Gemeinschaftskrankenhaus Herdecke (GKH), an academic teaching hospital of Witten/Herdecke University in Germany. The two main aims of the CEWIM are [7] (1) to promote ASP in clinical patient care while providing students with close supervision and (2) to help students learn and practice integrative medicine (in this case, to complement conventional medicine with anthroposophic medicine [8]). 

While most studies investigating ASP focus on its educational value for learners, few have explored its potential influence on the community of practice and on the learners' work, including quality of care. The findings of such studies have indicated that ASP may also be of benefit to patients, since they gain a better understanding of their disease [9] and are more satisfied that their needs have been met [10]. This raises the question whether ASP has an effect on patient centeredness, which the Institute of Medicine defines as “providing care that is respectful of and responsive to individual patient preferences, needs and values, and ensuring that patient values guide all clinical decisions” ([11], page 40). The aim of the present study was, therefore, to determine whether ASP has a certain impact on quality of care in an integrative inpatient setting. More specifically, we wished to compare the experiences of patients on the CEWIM to those of patients on the regular internal medicine wards at the GKH with respect to the following aspects of patient centeredness:

- physician-patient interaction,
- physician empathy, defined as a specific ability to communicate and help patients based on a deeper understanding of their situation, their perspectives, and their feelings [12],
- patient enablement, defined as the extent to which a patient feels empowered after a medical consultation, in terms of being able to cope with, understand, and manage their illness [13]).

## 2. Methods

### 2.1. Clinical Setting

The Department of Internal Medicine at the GKH, which offers the same diagnostic and interventional services as a regional hospital (including an intensive care unit), is divided into three different wards: one *short-term ward *with 20 beds, which is mainly for patients with fewer symptoms and less severe diseases (average stay: 4.5 days), and two *long-term wards* for patients with more complex and severe diseases. Particularly on these long-term wards, there are patients with specific interest in complementary medicine. One of the two long-term wards is more specialized in oncological and palliative care (26 beds; average stay: 9.9 days); the other is focused on gastroenterological and cardiological care (36 beds; average stay: 11.2 days). The CEWIM was incorporated into the latter medical ward, using 10 to 12 of the ward's 36 beds. On the CEWIM, four to five students take over the work of one house officer on the ward, meaning that together they are responsible for the care of a total of 10 to 12 patients. The students are supported and supervised by the house officer and a consultant. Important decisions, such as making changes to a patient's medication or arranging diagnostic interventions, may only be made after consultation and in agreement with the two supervising physicians. Students' care of the patients on the ward involves the usual diagnostic and therapeutic procedures performed on a conventional medical ward as well as complementary medicine being offered to interested patients. Based on a holistic view of the patient, students provide conventional and specific complementary treatments, such as natural remedies, rhythmic massage, external applications, art therapy, therapeutic eurythmy (a form of meditative movement), and biographic counseling [8]. Student rotations on the CEWIM are 16 weeks in duration and have taken place twice a year each year since 2009. 

### 2.2. Educational Setting

Originally launched as a pilot project in 2007, the CEWIM became a permanent program in 2009. The CEWIM is part of the Integrated Curriculum of Anthroposophic Medicine (ICURAM), an optional program in anthroposophic medicine that is integrated into the full six-year program of the regular undergraduate medical curriculum at Witten/Herdecke University [14]. The main goal of the ICURAM program is to emphasize a broader, multifaceted, and holistic view of human beings and to prepare students for patient-centered integrative and anthroposophic care. In order to achieve this goal, learner-centered educational strategies and learning objectives were developed in close contact and together with students. These strategies have been summarized into what is called the *ESPRI* ^2^ *T* approach, which combines exploratory learning, supported participation, patient-oriented learning, reflective practice, integrated learning, an integrative approach, and team-oriented learning [14]. The CEWIM was developed based on this approach. Apart from providing students with clinical training through ASP, it also offers students specific learning opportunities.

- Students learn how to integrate the different perspectives and treatment options of conventional medicine and complementary anthroposophic medicine and to appreciate the specific values and limitations of each approach.
- Students are involved in interprofessional learning by being fully integrated into the healthcare team and by working together with nurses and healthcare practitioners practicing art therapy, music therapy, therapeutic eurythmy, massage, physiotherapy, and psychotherapy. Interprofessional learning is enhanced by an interprofessional module (held during Week 1), weekly team meetings with therapists, and monthly meetings with the nursing team.
- Reflective practice is promoted through a clinical reflection training seminar [15] held every two weeks with a professional supervisor. During the seminar, students reflect on challenging situations faced during their rotation, such as interactions with patients, difficulties working with team members, and professional self-management issues. This seminar was developed because participating students had expressed the desire for the opportunity to process and discuss their sometimes difficult experiences and their professional development.

### 2.3. Research Instruments

The following instruments were used to evaluate and compare the perceived quality of care received by patients of the CEWIM and by patients of the regular “nonstudent” wards of the Department of Internal Medicine at the GKH.

- *The Picker Inpatient Questionnaire (PIQ).* Originally developed in the USA by Cleary et al., the PIQ was accredited in 1997 by the Joint Commission for Accreditation of Health Care Organizations [16, 17]. Patients surveyed using the PIQ are asked to report their experience with specific events and processes in the hospital. The questionnaire includes eight questions on patient-physician interaction as well as two general quality items, for example, asking patients whether they have recommended their ward to friends and relatives. For the purpose of our study, the German version of the PIQ was used. We also supplemented the PIQ with additional items in order to assess specific qualities of integrative care and anthroposophic medicine.
- *The Consultation and Relational Empathy (CARE) Questionnaire.* Developed by Mercer et al. [18] as a patient-reported outcome measure for physician empathy, the CARE contains 10 items, all beginning with the words “how was the doctor at...” (“really listening,” “making you feel at ease,” etc.). For the purpose of our study, the German version of the CARE measure was used. The original English version showed good internal validity (Cronbach's alpha: 0.92) and face validity [18]. Evaluation of the German version confirmed that the one-dimensional structure of the original English version had been replicated [19].
- *The Patient Enablement Instrument (PEI).* Developed by Howie et al. [13], this six-item questionnaire asks patients whether as a result of their visit to their doctor they feel they are able to understand their illness, help themselves, and so forth. Since we used the questionnaire for the evaluation of a clinical stay, we reworded the first part of each of these items to say “As a result of the treatment received at the hospital, do you feel you are....” The German version of the PEI was used in the study [20].
- Patients of the CEWIM were also asked to rate whether student participation on the ward had a positive, neutral, or negative impact on their care.

To ensure that medical students were included in the patients' assessments of the quality of care they received, the word “physician” was replaced with the phrase “the team of physicians” in all relevant questions. A note was also made that students should be considered a part of this team.

### 2.4. Sampling

To evaluate the perceived quality of care received on the CEWIM, a survey was conducted of all patients that had been treated by four cohorts of medical students between the fall of 2010 and the summer of 2012. All patients meeting the inclusion criteria (*n* = 215, see Table 1) were surveyed by anonymous written questionnaire within eight weeks after their discharge. Completed questionnaires were then sent back to the Picker Institute, Germany, where the data were sampled and a preliminary analysis was performed.

The perceived quality of care received by patients of the regular internal medicine wards (control group, *n* = 250) was assessed using results taken from the most recently conducted routine evaluation of the GKH. Every two years, the GKH undergoes an evaluation, the last of which was conducted in the fall of 2011 over a period of two months. The data collection procedure and the questionnaires used for the routine GKH evaluation are the same as those used for the CEWIM patient survey. 

### 2.5. Statistical Analysis

The patient characteristics of each patient group were calculated and compared using the Mann-Whitney *U* test. Only those characteristics for which a significant difference was found between both groups (age, education, interest in anthroposophic medicine, and number of nonphysician therapies obtained) were included as covariates in a univariate ANCOVA where the outcomes of the PIQ, the CARE, and the PEI were compared. IBM SPSS 20.0 was used for these analyses. A *P* value <0.05 was considered to indicate a significant statistical difference.

## 3. Results

Of the 215 CEWIM patients, 103 returned the questionnaire (response rate 47.9%), whereas in the control group, this was 94 out of 250 (37.6%). Patients of the CEWIM were, on average, six years younger (60.7 versus 66.6 years), had a better education (e.g., 20.6% versus 11.6% finalized tertiary education), were more often very interested in anthroposophic medicine (55% versus 29.6%), and obtained more often nonphysician therapies (59.8% versus 40.5%) than patients in the control group. No significant differences were found between the two patient groups for the other characteristics assessed, including sex, possession of private health insurance, duration of disease, and health status (see Table 1). 

**Figure 1:**
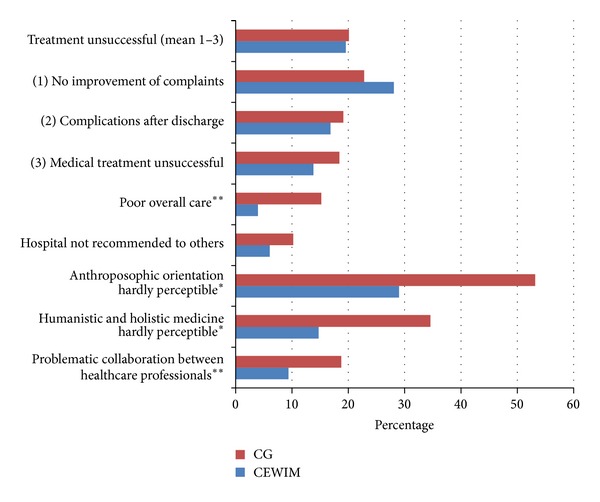
Frequency of problems with general aspects of integrative care as reported by CEWIM patients and the CG. Note that *indicates a significant difference between the CEWIM and the CG wards; analysis of covariance (ANCOVA), *P* < 0.05; **P ≤ 0.01.

**Figure 2:**
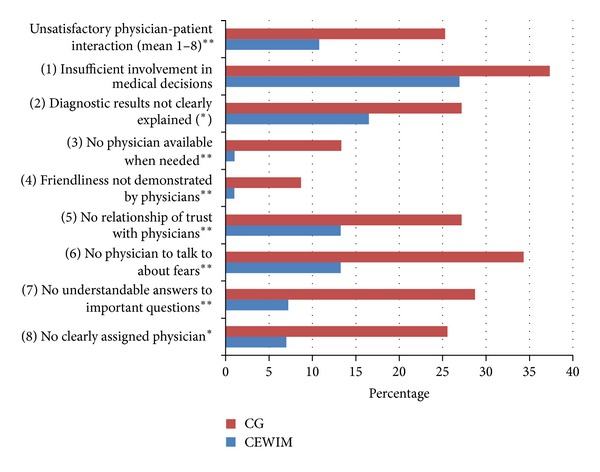
Frequency of problems with physician-patient interaction as reported by CEWIM patients and the CG. Note that * indicates a significant difference between the CEWIM and the CG wards; analysis of covariance (ANCOVA), *P* < 0.05; **P ≤ 0.01; *P < 0.1

While the results of the PIQ (see Figure 1) revealed no significant differences between the two types of patient wards in terms of the success of medical treatment, the CEWIM demonstrated significantly better results than the other wards with regard to the success of overall care and received higher ratings on six out of the eight physician-patient interaction items (see Figure 2) and also better CARE and PEI scores (see Table 2). These analyses were performed after controlling for all covariates showing a significant difference between the two patient groups (age, education, interest in anthroposophic medicine, and number of nonphysician therapies obtained).

**Figure 3:**
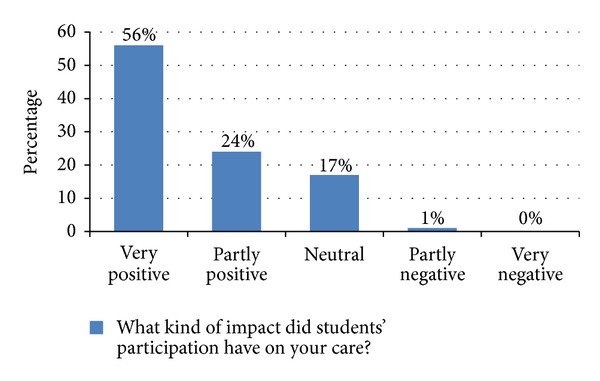
CEWIM patient assessments of student participation in clinical care.

Eighty Percent of the CEWIM patients reported that student participation on the ward had a somewhat positive or very positive impact on the quality of care they received. Only one percent felt it had a somewhat negative impact on the quality of care received. None considered it to have had a very negative impact (see Figure 3). 

## 4. Discussion

The aim of our study was to determine whether there is a difference in the quality of care experienced by patients on the CEWIM, where students actively participate in patient care, and wards of the same department where students are not involved in care. In particular, we were interested in whether ASP has an impact on certain qualities of patient centeredness, namely, patient-physician interaction in general, perceived physician empathy, and patient enablement.

The results of our study indicate that while ASP—as implemented on the CEWIM—has no significant impact on the success of medical treatment in terms of the improvement of complaints or the rate of reported complications after discharge, it does improve patient-physician interaction, physician empathy, and patient enablement. The CEWIM also fared better than the control group wards in other areas which are specific to integrative medicine; namely, the practice of holistic and humanistic medicine, a noticeable anthroposophic orientation, and collaboration between healthcare professionals. 

A preliminary study of the initial two-rotation pilot phase of the CEWIM [21] had found the patient-physician relationship to be rated better by patients on the CEWIM than by patients on the same medical ward at the GKH without ASP (control group 1) and by patients from conventional internal medicine wards in Germany (control group 2). However, only the difference between the CEWIM and control group 2 was found to be statistically significant. It was, therefore, impossible to determine whether the positive assessments of physician-patient interaction on the CEWIM were the result of ASP on the ward or whether they are merely attributable to the medical setting at the GKH. Exploration of additionally collected qualitative data did, however, indicate that care on the CEWIM was perceived as more holistic and individualized. 

By including a greater number of patients and students in the present study, it was possible to detect a significant difference between the CEWIM and the GKH medical wards without ASP, in particular with regard to certain qualities of patient centeredness. The results of our study seem surprising given that patients tend to prefer to be treated by experienced professionals rather than by students still in medical school. This preference is, of course, understandable, since students tend to be more uncertain of themselves, slower, overstrained at an earlier stage, and need more time and attempts to perform most procedures and practical tasks (e.g., inserting an IV catheter or performing a physical examination). It is also assumed that resident physicians and fully licensed physicians have greater practical knowledge and are better in other areas, such as time management, clinical thinking, and clinical decision making. 

Several hypotheses can be offered to explain why patients' ratings of patient centeredness were higher on the CEWIM.

1. *Organizational Advantages. *Since students are responsible for the care of fewer patients than regular physicians, they can dedicate more time to their patients. In addition, unlike physicians on the regular wards, students on the CEWIM do not have night shifts and are continuously on the ward. These two organizational advantages may be reflected in the better PIQ scores for “physician availability” and “having a clearly assigned physician.” Similarly, another study found time and physician availability to have a comparable positive effect on patients' assessments of physician empathy among patients with private health insurance [21].
2. *Specific Qualities of Student Communication with Patients. *The results of the CARE questionnaire suggest that patients on the CEWIM found students to be better at letting them tell their story, at really listening, and at being interested in them as a whole person. These aspects require not only dedicating more time to patients but also motivation, an open interest in another person, and a high level of commitment. These outcomes on empathy are supported by the better PIQ scores for “enough friendliness,” “relationship of trust,” and “possibility to talk about fears” in the CEWIM group. Moreover, patients on the CEWIM more frequently reported receiving “understandable answers to important questions,” which could be explained by the fact that students still tend to use more everyday language instead of medical terms.
3. *The Impact of Teaching and Supervision.* On the CEWIM, student learning is promoted through reflection and discussion on the daily problems and tasks associated with each of the students' patients. In addition to the usual ward rounds and seminars, students participate in weekly teaching rounds and integrative case conferences. These activities give students opportunities to dedicate greater focus to individual patient cases and find optimal solutions to their problems. In other words, they have more time to work through cases thoroughly and conscientiously than a regular physician would have. This may further be supported by the reflective practice module held every two weeks, which offers students opportunities to reflect on challenging interactions with their patients.
4. *Students' Impact on the Healthcare Team*. It is possible that, by asking relevant questions, students may encourage healthcare professionals to reassess and improve the usual procedures on the ward and to work with greater mindfulness and reflection. Being asked to act as a role model may also promote better and more comprehensive performance.

The results of our study correspond with those of other studies, which have found ASP to have a positive impact on patient care. A study by Coleman and Murray, for example, found that patients enjoyed being involved in a community-based teaching program, had acquired greater knowledge about their disease, had enhanced self-esteem, and felt that the service they received was better [9]. Patients treated on an interprofessional clinical education ward in London [10] indicated being more satisfied than patients on a conventional ward with the care they received. They felt that students had communicated better in terms of active listening and answering their questions. Greater motivation and enthusiasm on the part of students were also seen as a relevant factor contributing to higher satisfaction with the education ward. 

Although, as this study has shown, ASP plays a significant role in perceived quality of care, it is also vital to provide the proper learning environment that supports students during their participation on the ward. Various studies have shown that empathy and patient centeredness tend to decline during participation in clinical education and in residency [22]. While the reasons for this are still not fully clear, it seems that the process of adaptation to the contemporary culture of clinical practice may lead to a more effective and focused, yet less patient-oriented, attitude. Qualities of empathy and patient centeredness may therefore be stronger at the beginning of clinical education. Carmel and Glick, for example, analyzed the characteristics of compassionate and empathic physicians and found a significant correlation with younger age and fewer years of medical experience [23]. Studies on residents, on the other hand, have reported higher levels of stress, depression, and burnout in residents [24, 25], all of which lead to less patient-centered and empathic interaction with patients. A qualitative study from Belgium describes patient centeredness as being negatively affected by tiredness and perceived time pressure, by nonpatient-centered role models, and by feeling overwhelmed by powerful experiences [26]. Thanks to the learning environment provided on the CEWIM, medical students still experience a sense of security and assurance while working under close supervision; the additional attendance and perceived learned-centered approach may make it easier for them to practice patient-centered care. 

### 4.1. Strength and Limitations

Both the CEWIM patient group and the control group were comparable in size (103 versus 94 patients), sex, insurance status, health status, and duration of disease. A possible explanation for the lower response rate of the control group compared to the CEWIM patient groups (38% versus 48%) may be that this group had more older age patients and more patients with dementia, making it more difficult for them to complete a written survey. The fact that more of the CEWIM patients were satisfied with their care may have also contributed to a higher response rate. The lower percentage of patients in the control group with an interest in anthroposophic medicine reflects the fact that two of the three internal medicine wards at the GKH offer a greater range of complementary therapies. The “short-term ward” does not offer so many art therapies and other nonphysician therapies. The number of patients with an interest in anthroposophic medicine was therefore higher on the CEWIM than on the control group wards, one of the reasons why it was important to control for different patient characteristics as part of our study.

Since the long-term wards have more patients with a specific interest in anthroposophic medicine, it may be that the resulting higher amount of patients obtaining more complementary nonphysician therapies led to greater satisfaction with the CEWIM. However, inclusion of the patient characteristic “number of nonphysician therapies obtained” as a covariate did not change the significance of the differences in quality of care found between the two patient groups. Furthermore, 80% of the CEWIM patients rated the impact of student participation on their care as positive while only one out of the 103 CEWIM patients reported it to have a somewhat negative influence.

## 5. Implications for Future Research 

The results of this study suggest that ASP has a positive effect on perceived patient centeredness. Further studies are needed to determine whether similar results can be observed in other contexts, such as in other clinical specialties or in conventional hospitals. A comprehensive economic analysis is also desired to assess cost intensiveness of the CEWIM compared to a regular ward. Also, more research is necessary to analyze the specific conditions of the learning environment enabling a positive influence of ASP on the quality of care. 

## 6. Conclusion

Our study shows that ASP may enhance patient-physician interaction, physician empathy, and patient enablement. In addition, our findings suggest that the relationship between students and the community of practice is not just a one-way street. Active student participation in the community is of benefit both to students and to the healthcare team and patients. Student participation and the establishment of a favorable and supportive learning environment may therefore be considered factors which can be used to improve the care provided by future healthcare teams practicing integrative medicine. 

## Figures and Tables

**Table 1:** Patient characteristics.

	CEWIM	Control group (CG)
Number of patients	234	494
Exclusion criteria		
Died	2	17
<18 years	0	0
<2-night stay	9	80
Readmission	4	50
Other	4	22
Sum of patients excluded	19	169
Patients contacted	215	250^1^
Questionnaires returned	103 (47.9%)	94 (37.6%)

Sex (female)	65 (63.1%)	56 (60.9%)
Age (mean, SD)*	60.7 ± 17.3 years	66.6 ± 16.3 years
Private health insurance	22 (21.8%)	19 (20.4%)

Education*		
Primary education	3 (2.9%)	2 (2.3%)
Lower secondary education	12 (11.8%)	14 (16.3%)
Lower sec. ed. + apprenticeship	30 (29.4%)	33 (38.4%)
Lower sec. ed. + postsecondary education	19 (18.6%)	23 (26.7%)
Upper secondary education	17 (16.7%)	4 (4.7%)
Tertiary education (university)	21 (20.6%)	10 (11.6%)
Duration of disease		
6–12 months	21 (32.3%)	16 (29.1%)
1–3 years	7 (10.8%)	13 (23.6%)
3–5 years	9 (13.8%)	5 (9.1%)
>5 years	28 (43.1%)	21 (38.2%)
AM important for choice of hospital*		
Yes, very much	55 (55.0%)	24 (29.6%)
Yes, somewhat	20 (20.0%)	16 (44.4%)
No	25 (25.0%)	41 (50.6%)
Health status		
Poor	20 (19.8%)	15 (16.5%)
Moderate	50 (49.5%)	44 (48.4%)
Good	21 (20.8%)	29 (31.9%)
Very good	10 (9.9%)	2 (2.2%)
Excellent	0 (0%)	1 (1.1%)
Number of nonphysician therapies obtained^∗2^		
No	41 (40.2%)	50 (59.5%)
1–3	45 (44.1%)	21 (25.0%)
≥4	16 (15.7%)	13 (15.5%)

^1^Randomly drawn from the sample of 325 patients remaining after exclusion.

^2^These therapies include art therapy, music therapy, clay modeling, speech therapy, therapeutic eurythmy, rhythmic embrocations, massage, physiotherapy, and psychological counseling.

*indicates a significant difference between CEWIM patients and controls; Mann-Whitney *U* test, *P* < 0.05.

**Table 2:** Differences of patient groups in physician patient interaction, empathy (CARE), and patient enablement (PEI).

Dependent variables		Mean	SD	Analysis of covariance (ANCOVA)
Empathy (CARE)^a^	CEWIM	1.46	(±0.55)	*F* = 16.096*P* < 0.001
	Controls	1.83	(±0.90)	

Patient enablement (PEI)^b^	CEWIM	0.95	(±0.64)	*F* = 5.237*P* = 0.024
	Controls	0.68	(±0.63)	

^a^Low values indicate high empathy.

^b^High values indicate high enablement.

## References

[1] Lave J, Wenger E (1991). *Situated Learning: Legitimate Peripheral Participation*.

[2] Wenger E (1999). *Communities of Practice: Learning, Meaning, and Identity*.

[3] Dornan T, Boshuizen H, King N, Scherpbier A (2007). Experience-based learning: a model linking the processes and outcomes of medical students’ workplace learning. *Medical Education*.

[4] Remmen R, Denekens J, Scherpbier A (2000). An evaluation study of the didactic quality of clerkships. *Medical Education*.

[5] Remmen R, Derese A, Scherpbier A (1999). Can medical schools rely on clerkships to train students in basic clinical skills?. *Medical Education*.

[6] Schrauth M, Weyrich P, Kraus B, Jünger J, Zipfel S, Nikendei C (2009). Workplace learning for final-year medical students: a comprehensive analysis of student’s expectancies and experiences. *Zeitschrift für Evidenz, Fortbildung und Qualitat im Gesundheitswesen*.

[7] Scheffer C, Edelhäuser F, Tauschel D, Riechmann M, Tekian A (2010). Can final year medical students significantly contribute to patient care? A pilot study about the perception of patients and clinical staff. *Medical Teacher*.

[8] Heusser P, Scheffer C, Neumann M, Tauschel D, Edelhäuser F (2012). Towards non-reductionistic medical anthropology, medical education and practitioner-patient-interaction: the example of Anthroposophic Medicine. *Patient Education and Counseling*.

[9] Coleman K, Murray E (2002). Patients’ views and feelings on the community-based teaching of undergraduate medical students: a qualitative study. *Family Practice*.

[10] Reeves S, Freeth D, McCrorie P, Perry D (2002). “It teaches you what to expect in future...”: interprofessional learning on a training ward for medical, nursing, occupational therapy and physiotherapy students. *Medical Education*.

[11] Institute of Medicine (2001). *Crossing the Quality Chasm: A New Health System for the 21st Century*.

[12] Mercer SW, Reynolds WJ (2002). Empathy and quality of care. *The British Journal of General Practice*.

[13] Howie JGR, Heaney DJ, Maxwell M, Walker JJ, Freeman GK, Rai H (1999). Quality at general practice consultations: cross sectional survey. *British Medical Journal*.

[14] Scheffer C, Tauschel D, Neumann M (2012). Integrative medical education: educational strategies and preliminary evaluation of the Integrated Curriculum for Anthroposophic Medicine (ICURAM). *Patient Education and Counseling*.

[15] Lutz G, Edelhäuser F, Scheffer C, Tauschel D, Neumann M “Without it, it would have been much worse”: a mixed-method evaluation of clinical reflective practice in integrative care education.

[16] Cleary PD, Edgman-Levitan S, Roberts M (1991). Patients evaluate their hospital care: a national survey. *Health Affairs*.

[17] Jenkinson C, Coulter A, Bruster S (2002). The picker patient experience questionnaire: development and validation using data from in-patient surveys in five countries. *International Journal for Quality in Health Care*.

[18] Mercer SW, Maxwell M, Heaney D, Watt GCM (2004). The consultation and relational empathy (CARE) measure: development and preliminary validation and reliability of an empathy-based consultation process measure. *Family Practice*.

[19] Neumann M, Wirtz M, Bollschweiler E, Warm M, Wolf J, Pfaff H (2008). Psychometric evaluation of the German version of the “consultation and relational empathy” (CARE) measure at the example of cancer patients. *Psychotherapie Psychosomatik Medizinische Psychologie*.

[20] Neumann M, Schwarzkamp U, Pfaff H (2006). The Patient Enablement Instrument (PEI) as an economic and valid outcome in psycho-oncological care? Psychometric characteristics of the German version of the PEI in a first explorative study. *Psycho-Oncology*.

[21] Neumann M, Bensing J, Wirtz M (2011). The impact of financial incentives on physician empathy: a study from the perspective of patients with private and statutory health insurance. *Patient Education and Counseling*.

[22] Neumann M, Edelhäuser F, Tauschel D (2011). Empathy decline and its reasons: a systematic review of studies with medical students and residents. *Academic Medicine*.

[23] Carmel S, Glick SM (1996). Compassionate-empathic physicians: personality traits and social-organizational factors that enhance or inhibit this behavior pattern. *Social Science and Medicine*.

[24] Rosen IM, Gimotty PA, Shea JA, Bellini LM (2006). Evolution of sleep quantity, sleep deprivation, mood disturbances, empathy, and burnout among interns. *Academic Medicine*.

[25] Shanafelt TD, Bradley KA, Wipf JE, Back AL (2002). Burnout and self-reported patient care in an internal medicine residency program. *Annals of Internal Medicine*.

[26] Bombeke K, Symons L, Debaene L, De Winter B, Schol S, Van Royen P (2010). Help, I’m losing patient-centredness! Experiences of medical students and their teachers. *Medical Education*.

